# Chemo-Predictive Assay for Targeting Cancer Stem-Like Cells in Patients Affected by Brain Tumors

**DOI:** 10.1371/journal.pone.0105710

**Published:** 2014-08-21

**Authors:** Sarah E. Mathis, Anthony Alberico, Rounak Nande, Walter Neto, Logan Lawrence, Danielle R. McCallister, James Denvir, Gerrit A. Kimmey, Mark Mogul, Gerard Oakley, Krista L. Denning, Thomas Dougherty, Jagan V. Valluri, Pier Paolo Claudio

**Affiliations:** 1 Department of Biochemistry and Microbiology, Joan C. Edwards School of Medicine, Marshall University, Huntington, West Virginia, United States of America; 2 Translational Genomic Research Institute, Marshall University, Huntington, West Virginia, United States of America; 3 Department of Neurosurgery, Joan C. Edwards School of Medicine, Marshall University, Huntington, West Virginia, United States of America; 4 Department of Medical Oncology, St. Mary's Hospital, Huntington, West Virginia, United States of America; 5 Department of Pediatrics, Joan C. Edwards School of Medicine, Marshall University, Huntington, West Virginia, United States of America; 6 Department of Pathology, Joan C. Edwards School of Medicine, Marshall University, Huntington, West Virginia, United States of America; 7 Department of Biology, Marshall University, Huntington, West Virginia, United States of America; 8 Department of Surgery, Joan C. Edwards School of Medicine, Marshall University, Huntington, West Virginia, United States of America; Institute of Clinical Physiology, c/o Toscana Life Sciences Foundation, Italy

## Abstract

Administration of ineffective anticancer therapy is associated with unnecessary toxicity and development of resistant clones. Cancer stem-like cells (CSLCs) resist chemotherapy, thereby causing relapse of the disease. Thus, development of a test that identifies the most effective chemotherapy management offers great promise for individualized anticancer treatments. We have developed an ex vivo chemotherapy sensitivity assay (ChemoID), which measures the sensitivity of CSLCs as well as the bulk of tumor cells to a variety of chemotherapy agents. Two patients, a 21-year old male (patient 1) and a 5-month female (patient 2), affected by anaplastic WHO grade-III ependymoma were screened using the ChemoID assay. Patient 1 was found sensitive to the combination of irinotecan and bevacizumab, which resulted in a prolonged disease progression free period of 18 months. Following recurrence, the combination of various chemotherapy drugs was tested again with the ChemoID assay. We found that benzyl isothiocyanate (BITC) greatly increased the chemosensitivity of the ependymoma cells to the combination of irinotecan and bevacizumab. After patient 1 was treated for two months with irinotecan, bevacizumab and supplements of cruciferous vegetable extracts containing BITC, we observed over 50% tumoral regression in comparison with pre-ChemoID scan as evidenced by MRI. Patient 2 was found resistant to all treatments tested and following 6 cycles of vincristine, carboplatin, cyclophosphamide, etoposide, and cisplatin in various combinations, the tumor of this patient rapidly progressed and proton beam therapy was recommended. As expected animal studies conducted with patient derived xenografts treated with ChemoID screened drugs recapitulated the clinical observation. This assay demonstrates that patients with the same histological stage and grade of cancer may vary considerably in their clinical response, suggesting that ChemoID testing which measures the sensitivity of CSLCs as well as the bulk of tumor cells to a variety of chemotherapy agents could lead to more effective and personalized anticancer treatments in the future.

## Introduction

Although ependymomas are the third most common type of brain tumor in children (following astrocytoma and medulloblastoma), they are relatively rare, with approximately 200 cases diagnosed in the US each year [1], [2]. They account for 60% of all intramedullary tumors and 50% arise in the filum terminale [3].

The treatment of ependymomas can be challenging. The initial standard treatment for ependymoma is surgery often followed by radiation therapy, and chemotherapy. Although chemotherapy has been used extensively in children with ependymomas, there is little clinical evidence that chemotherapy improves survival of children with this type of tumor. Chemotherapy is often reserved for patients with residual tumor after surgery and for children younger than 3 years of age in an attempt to delay radiation therapy [4].

It is not entirely clear why there is not an improved survival with chemotherapy, but it is known that resistance to a variety of commonly used chemotherapeutic agents is common in ependymoma [5]. Therefore investigation and development of novel strategies and integrated therapies are required to find more effective treatments for this type of tumor.

Patients with the same stage and grade of cancer may vary considerably in their clinical response and toleration of chemotherapy. Ineffective anticancer therapy can result in unnecessary toxicity and the development of resistant clones. The surviving cancer cells are often more resistant to therapy. Many attempts have been made over the years to develop an *ex-vivo* anti-cancer test that could help discern the best treatment options for each individual patient while minimizing toxicity.

Animal xenograft models have shown that only a subset of cancer cells within each tumor is capable of initiating tumor growth. This capability has been shown in several types of human cancers, to include ependymomas [6]. This pool of cancer cells is operationally defined as the “Cancer Stem-Like Cell” (CSLC) subset. According to the “cancer stem-like cell” theory, tumors are a complex, growing population of abnormal cells originating from a minority of CSLCs. These cells maintain stem-like characteristics in that they proliferate very slowly and have an inherent capacity to self-renew and differentiate into phenotypically heterogeneous, aberrant progeny [7]–[10]. Unlike the bulk of tumor cells, CSLCs resist chemotherapy and radiation therapy and are responsible for tumor relapse and metastasis [9], [10].

Some ependymomas express various markers of stemness, including CD133. In addition, relapsed tumors exhibit a gene expression signature constituted by up-regulated genes involved in the kinetochore (ASPM, KIF11) or in neural development (CD133, Wnt and Notch pathways) [11].

Targeting CSLCs in addition to the bulk of other cancer cells within a tumor is a new paradigm in cancer treatment. Our recent studies show that a Hydrodynamic Focusing Bioreactor (HFB) (Celdyne, Houston TX) selectively enriches CSLCs from cancer cell lines that can be used in a chemosensitivity assay [8]. Further, using this strategy we optimized the enrichment of CSLCs from tumor biopsies and have developed the ChemoID chemotherapy sensitivity assay, which measures the response of CSLCs and the bulk of tumor cells to chemotherapy to determine the most effective combination of anticancer drugs for malignant tumors of the nervous system.

In this study we report, for the first time, our investigation using the ChemoID assay to measure the sensitivity and resistance of CSLCs and bulk of tumor cells cultured from 2 biopsies of human ependymoma challenged with several chemotherapy agents which were also correlated to the response of animal xenografts treated with the predicted drugs and to the clinical response of the treated patients.

## Materials and Methods

### Reagents

Benzyl isothiocyanate (BITC) was purchased from Sigma Chemical Co. (St. Louis, MO). Bevacizumab (Avastin), Cisplatin, Oxaliplatin, Arabinoside-C, VP-16, Irinotecan (Camptosar, CPT-11), Busulfan, Methotrexate, were acquired as clinical grade chemotherapy agents.

### Patients

Case 1 is a 21-year-old male patient diagnosed with intradural, intramedullary, and extramedullary anaplastic diffuse spinal ependymoma, WHO grade III. Case 2 is a 5-month old female patient diagnosed with anaplastic WHO grade III ependymoma.

ChemoID assay was performed after obtaining patient's written informed consent in accordance with the ethical standards of the Helsinki Declaration (1964, amended most recently in 2008) of the World Medical Association. Any information, including illustrations, has been anonymized. Marshall University Institutional Review Board (IRB) has approved this research under the protocol #326290. Participants or guardians of participant (in case of a child participant) provided their written consent on an IRB approved informed consent form to participate in this study after being educated about the research protocol. Ethics committees/IRB at Marshall University approved this consent procedure. For Children participants to the study, written informed consent was obtained from the next of kin, caretakers, or guardians on behalf of the minors/children enrolled in your study.

### Single Cell Suspension and Primary Cell Culture

Single-cell suspensions from the ependymoma biopsies were prepared using the gentleMACS Dissociator (Miltenyi, Auburn, CA), and C Tubes using a standardized, semi-automated protocol based on a combination of mechanical tissue disruption and incubation with a 50% solution 0.025% trypsin and Accutase (Innovative Cell Technologies, San Diego, CA). Cells were serially plated in 24-well, 12-well, 6-well, 10-cm treated dishes and cultured to subconfluence in RPMI-1640 medium supplemented with 5% irradiated, heat inactivated, defined fetal bovine serum (Thermofisher/Hyclone), and 50 U of penicillin and 5 µg of streptomycin/mL of medium (Thermofisher/Mediatech).

### Three-Dimensional Bioreactor CSLCs Culture

A hydrodynamic focusing bioreactor (HFB) (Celdyne, Houston TX) was used as previously described to selectively proliferate CD133(+) cancer stem-like cells [8]. Culture media, oxygenation, speed, temperature and CO_2_ were kept consistently constant for ten days.

Cells were counted and 1×10∧6 cells were placed in the rotating vessel set at 25 rpm with airflow set at 20%. Cells were then removed and counted again using trypan blue exclusion to determine cellular viability and cell number and plated in 96 wells for chemosensitivity testing. The cells were also incubated with florescent antibodies for phenotypic characterization [8].

### Cell Sorting

Up to 1×10∧7 cells were sorted by a magnetic-activated cell sorting (MACS) system, which consists of magnetic beads conjugated to an antibody against CD133 (Miltenyi, Auburn, CA). In brief, cells were harvested using 0.25% trypsin, pelleted and labeled with CD133/1 biotin and CD133/2-PE. Cells were washed and labeled with anti-biotin magnetic beads, and then passed through a magnetic column where CD133(+) cells were retained, while unlabelled cells passed through the column. The CD133(+) retained cells were eluted from the columns after removal from the magnet. Positive and negative cells were then analyzed by FACS for purity.

### Flow Cytometry Studies

Cells were analyzed by the antigenic criteria using anti-CD34 (Milteny Biotech, Auburn, CA), -CD38 (Milteny Biotech, Auburn, CA), -CD44 (BD Bioscience, Sparks, MD), -CD117 (Milteny Biotech, Auburn, CA), -CD133/2 (prominin1) (Milteny Biotech, Auburn, CA), -Oct3/4 (BD Bioscience, Sparks, MD), and –Nanog (BD Bioscience, Sparks, MD). Briefly, cells were detached using 0.02% EDTA in PBS and pelleted (10 min at 1,000 rpm), washed in 0.1% BSA in 1X PBS at 4°C and incubated in a solution of 1 mg antibody +9 mL 0.1% BSA in 1X PBS. Cells were washed in the same solution once and were analyzed using a C6 Accuri flow cytometer (BD Biosciences, San Jose, CA).

### ChemoID Assay

Sensitivity to chemotherapy was assessed using a viability assay (WST8) on 1×10∧3 cells plated in 5 replicas into 96-well plates. Briefly, equal number of bulk of tumor cells grown in monolayer and CSLCs grown in the bioreactor, were counted and seeded separately in 96-well dishes and incubated at 37°C for 24-hours. The cells were then challenged for a 1-hour pulse with a panel of anticancer drugs as chosen by the oncologist to mimic the average clinical chemotherapy infusion schedule.

To study the effect of BITC on chemosensitization of cancer cells to chemotherapy drugs, the cells were treated with an hour pulse 5–30 µM BITC followed by an hour of the various anticancer drugs. Each anticancer drug was tested in a range of doses including the clinically relevant dose.

A WST8 assay was performed 48-hours following chemotherapy treatment to assess cell viability as previously described [12]. A dose response chart was developed in which samples were scored as responsive (0–30% cell survival), intermediate (30–60% cell survival), and non-responsive (60–100% cell survival).

### Limiting Dilution Tumorigenic Assay in Immune Deficient Mice

A range of 1×10∧2, 1×10∧3, 1×10∧4, and 1×10∧5 ependymoma cells from Patient 1 were injected subcutaneously in 5 athymic immunodeficient nude^nu/nu^ mice per group. Briefly, an equal number of parental bulk of tumor cells grown in 2D monolayer, CD133(+) three-dimensionally grown in the hydrofocusing bioreactor, and CD133(+) MACSorted CSLCs were injected with 100 µL of matrigel in the flank of NOD-*Scid* mice and compared to the growth of CD133 negative cells for 3 months.

### Chemotherapy Animal Study

All animal studies have been conducted following approval from the Marshall University IACUC, protocol #373017. The effects of chemotherapies screened in vitro by the ChemoID assay was tested on human tumor biopsies that were xenografted in the flank of a NOD-*Scid* mouse model. 1×10∧6 ependymoma cells were mixed to 100 µL of matrigel (BD Biosciences, San Jose, CA) injected subcutaneously in the flank of 10 athymic, NOD.Cg-Prkdc *Scid* ll2rgtm1wjl/SzJ immunodeficient mice (NOD-*Scid*)/group and were grown for 10 weeks or until 100 mm∧3. Mice were randomized in different treatment and control groups and chemotherapy was administered by intraperitoneal (i.p.) injections in 200 µL as follows in a period of 4 weeks: 1) **Group #1**, Control group with primary tumor cells injected into flank and receiving i.p. sterile saline injections. **Group #2**, Experimental group injected i.p. with the least effective chemotherapy as determined by the *in vitro* ChemoID assay. **Group #3**, Experimental group injected i.p. with the most effective chemotherapy as determined by the *in vitro* ChemoID assay. **Group #4**, Experimental group injected i.p. with the second most effective chemotherapy as determined by the *in vitro* ChemoID assay. **Group #5**, Experimental group injected i.p. with the most effective combinatorial chemotherapy as determined by the *in vitro* ChemoID assay.

Chemotherapy mouse doses were calculated using a body surface area (BSA) normalization method [13] from the clinical dose and verified according to doses previously determined by a literature search.

### Euthanasia

Animals were euthanized following the current guidelines established by the latest Report of the AVMA Panel on Euthanasia using CO2 inhalation and asphyxiation followed by cervical dislocation.

### Statistical Analysis

Statistical analysis was performed using the IBM SPSS statistical software. The results for each variant in the different experimental designs represent an average of 3 different experiments. The data of 5 measurements were averaged; the coefficient of variation among these values never exceeded 10%. Mean values and standard errors were calculated for each point from the pooled normalized to control data. Statistical analysis of the significance of the results was performed with a 1-way ANOVA. *p* values of less than 0.05 were considered statistically significant.

## Results

### Patient 1 History and Selection of Chemotherapies with ChemoID Assay

**Figure 1:**
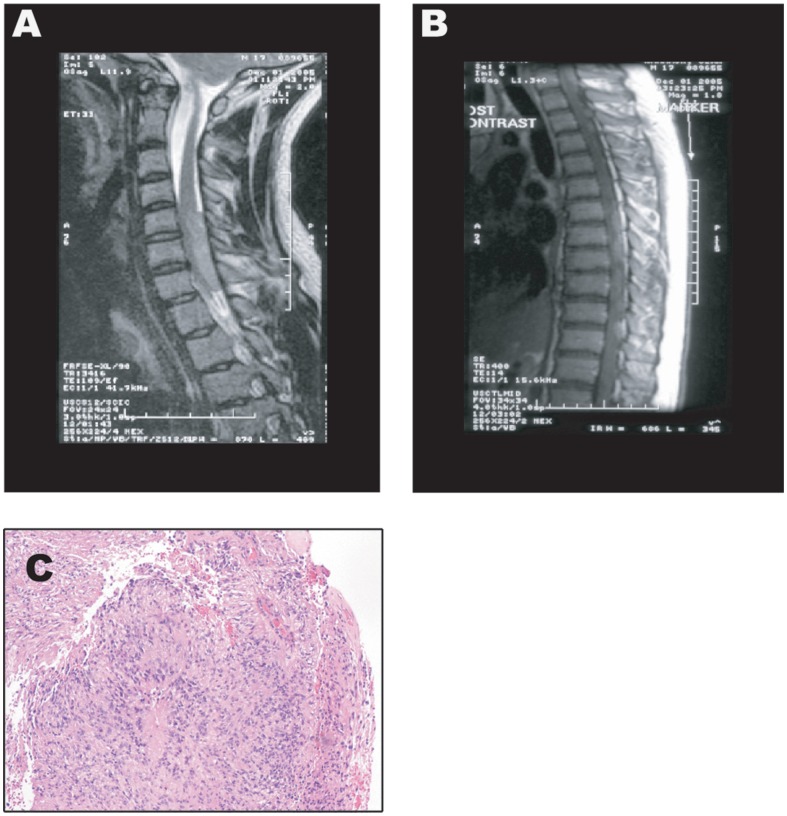
MRI Images and H&E Staining of the Anaplastic Ependymoma Case at Presentation. **A**) Magnetic Resonance Imaging (MRI) of the cervical spine showing the presence of an enhancing mass, which extends from mid C5 to inferior C7 (4.5 in length×1.0×2.0 in cephalocaudal and anteroposterior dimension) and causing cord compression. **B**) MRI of the thoracic spine showing an enhancing lesion at T2–3 (1.5 in length×0.6×0.6 cm in anteroposterior and transverse dimension) with several other smaller nodular masses, best seen on the T2 weighted sequence, which extended throughout the thoracic level to T11. **C**) Hematoxylin and Eosin staining of a tumor section showing an overall predominant dense cellular component, with primitive nuclear features, mitotic activity, necrosis and vascular proliferation. The presence of well formed, obvious perivascular pseudorosettes (with vasocentric pattern, perivascular nuclear-free zones, and classic thin glial processes radiating to/from the vessel wall) were found supportive of the diagnosis of intradural, extramedullary anaplastic diffuse spinal ependymoma, WHO grade III.

A physically active 17-year-old male presented in October 2005 with paresthesia in his feet and a rather severe perceptive loss. This became progressively worse in December 2005 going up his legs with rather severe numbness in the right leg and pain in his left leg, from the mid thigh down to the mid calf medially. On examination he had no focal weakness throughout his upper and lower extremities. He had hypoalgesia with partial sensory level in the upper thoracic spine down. He also had severe proprioception loss in his feet and toes. Magnetic resonance imaging (MRI) of the cervical spine showed the presence of an abnormal enhancing mass, which extended from mid C5 to inferior C7 (4.5 in length×1.0×2.0 in cephalocaudal and anteroposterior dimension) that caused cord compression (**Figure 1A**). MRI of the thoracic spine showed an enhancing lesion at T2–3 (1.5 in length×0.6×0.6 cm in anteroposterior and transverse dimension) with several other smaller nodular masses, best seen on the T2 weighted sequence, which extended throughout the thoracic level to T11 (**Figure 1B**).

The patient received a laminectomy in December 2005 at C5, C6, and C7 with partial resection of the tumor under microscope using microsurgical techniques. Following surgery, the patient was treated with radiation and temozolomide.

Morphological analysis of the histology sections stained with Hematoxylin & Eosin showed an overall predominant dense cellular component, with primitive and pleomorphic nuclei, increased mitotic rate and apoptosis, and foci with microvascular proliferation. The presence of well formed, obvious perivascular pseudorosettes (with vasocentric pattern, perivascular nuclear-free zones, and classic thin glial processes radiating to/from the vessel wall) were found to supporting the diagnosis of anaplastic diffuse spinal ependymoma, WHO grade III. **Figure 1C** shows the hematoxylin and eosin staining of a tumor section at diagnosis in 2005.

Sections of the tumor were evaluated by immunoperoxidase techniques with appropriate staining control sections. The tumor showed positive staining with antibodies to neuron specific enolase, vimentin, S-100, and GFAP. Weak staining occurred with the antibodies against actin. Focal staining occurred with antibodies to epithelial membrane antigen, cytokeratin AE1/AE3, and synaptophysin. The tumor was negative for leukocyte common antigen, desmin, and myogenin. In addition, a section stained with PAS showed a focal PAS-positive fibrillar material. Sections and tumor block were also sent to the Biopathology Center (BPC) of the Children's Oncology Group (COG) were two neuropathologists independently reviewed the case and confirmed the diagnosis of anaplastic ependymoma, WHO grade III.

**Figure 2:**
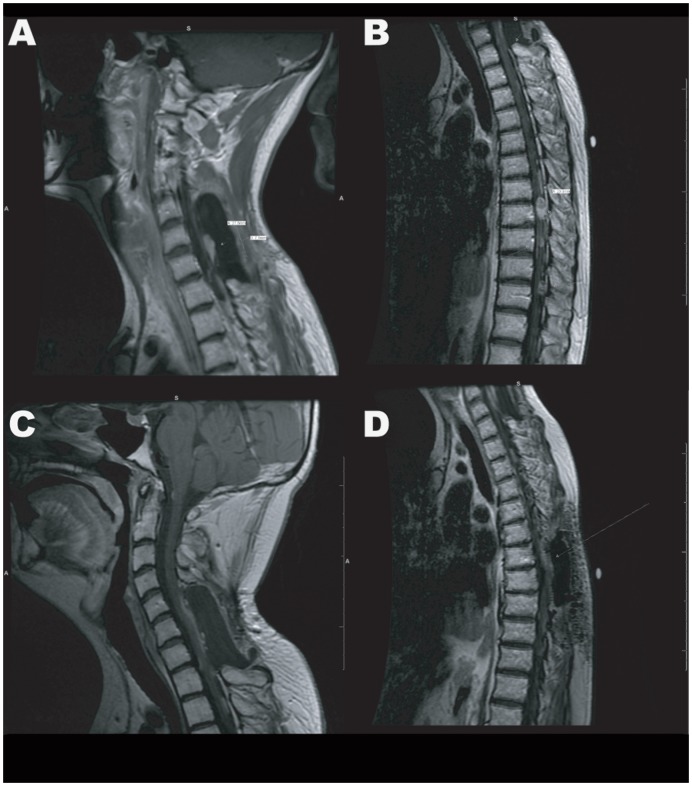
MRI Images of Cervical and Thoracic Spine. **A**) 2009 MRI of the cervical spine showing recurrence in the surgical area. **B**) 2009 MRI of the thoracic spine showing progression of the main lesion measuring 23.9 mm, and the appearance of several other smaller lesions. **C and D**) 2010 MRI of the cervical and thoracic spine showing tumor regression following a treatment with irinotecan and bevacizumab.

Following recurrence and progression, the patient received complex chemotherapy regimen in January 2006 and March 2006 with cyclophosphamide, thalidomide, celecoxib followed by etoposide, thalidomide and celecoxib. Chemotherapy treatment was concluded in September of 2006, but in August of 2007 patient had tumor regrowth at T7–T8 for which he underwent robotic radiosurgery treatment. The patient had another debulking surgery in April of 2008, but later in December of 2008 he had progressive numbness in his legs along with back pain with MRI showing recurrence in the surgical area (**Figure 2A**) as well as the lumbar spine. He was then treated again with temozolomide, but had no response to treatment.

In March 2009 because of progression of the disease he had a thoracic laminectomy and resection of the intradural intramedullary tumor. He had severe spinal compression and began having weakness in his legs. Due to further recurrence, the patient then had another debulking surgery in July of 2009. He also received oxaliplatin and etoposide treatment in July and August 2009, but the tumor progressed even more (**Figure 2B**).

Appropriate informed consent was signed and at the time of the debulking surgery of July 2009, a sterile biopsy was taken to assess the sensitivity of the tumor cells (bulk of tumor and CSLCs) toward standard-of-care chemotherapy drugs using our ChemoID assay. The biopsy was placed in RPMI-1640 sterile media and tissue was dissociated in our laboratory into a single-cell suspension with the use of a GentleMACS tissue dissociator (Miltenyi, Aubourn, CA). The single-cell ependymoma suspension was plated in RPMI-1640 in the presence of 5% irradiated, heat inactivated, defined fetal bovine serum, streptomycin and penicillin and cells were cultured as a monolayer for 15 days. Cells were immunophenotyped by flow cytometer using antibodies against CD34, CD38, CD44, CD117, CD133, OCT3/4, and Nanog.

The ependymoma cells were found positive to OCT3/4 (2.73%), Nanog (0.95%), CD133 (49.93%), CD117 (36.81%), and CD44 (20.39%) when compared to an isotype control antibody (**Figure S1 A-E**). A double staining of CD34 and CD38 showed the presence of 1.88% of the cells CD34+/CD38+, and 78.4% CD34+/CD38- cells (**Figure S1 F**).

**Figure 3:**
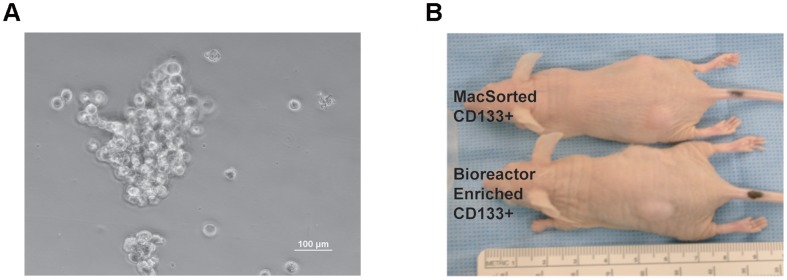
CD133 (+) Cells Grown in a Hydrofocusing Bioreactor form Xenografts in nude Mice. **A**) Contrast phase image of a cluster of enriched CSLCs following 7-days of culture in a hydrofocusing bioreactor. **B**) Immunodeficient nude mice (nu/nu) injected with 1×10∧2 ependymoma cells MacSorted CD133(+) cells or CD133(+) ependymoma cells grown in the hydrofocusing bioreactor, with the aid of 100 µL of matrigel in the flank formed a tumor within 3 months compared to CD133(−) cells.

To expand the CSLC population of CD133+ cells from the ependymoma primary culture, the ependymoma cells were cultured as previously described [8]. 1×10∧6 of the ependymoma cells from a monolayer primary culture were grown for ten days using Hydrodynamic Focusing Bioreactor (HFB) (Celdyne, Houston, TX) [8]. The ependymoma cells cultured in the bioreactor formed cell clusters (**Figure 3A**) which were expanded 14.7 fold (**Table 1**) and appeared to be 95.93% CD133 positive after 10 days of culture in the bioreactor (**Figure S1 C, enriched CSLCs**).

**Table 1:** Enrichment of CD133+ CSLCs using a hydrofocusing bioreactor.

	CD133+ cells	CD133- cells
**Day 0**	255,000	245,000
**Day 7**	3,748,500	159,036
**Fold**	14.7	−1.54

To verify the tumor initiating capacity of the HFB grown cells, we injected 5 immune deficient nude mice/group a range of 1×10∧2, 1×10∧3, 1×10∧4, and 1×10∧5 cells grown in the HFB (∼96% CD133+) and compared their growth to an equal number of CD133(+) MACsorted cells and CD133(−) cells for 3 months. We observed that both 1×10∧2 MacSorted CD133(+) cells or the CD133(+) from the bioreactor grew in all the immune deficient mice injected and formed a palpable tumor within 12 weeks (**Figure 3B**).

To perform the ChemoID assay a comparable number of cells (1×10∧5) bulk of tumor cells grown as a 2D monolayer and CSLCs enriched in the bioreactor [8] were separately plated into 96 wells plates (n-5 replicas) and were treated for an hour with a series of anticancer drugs at a range of concentrations including the clinically relevant dosage (**Table 2**). ChemoID assay was performed using a panel of drugs comprising of cisplatin, oxaliplatin, arabinoside-C, VP-16, busulfan, methotrexate, irinotecan, and bevacizumab as chosen by the treating oncologist.

**Table 2:** Clinical dose and calculated in vitro doses of the various chemotherapies.

	Bevacizumab	Cisplatin	Oxaliplatin	Arabinoside-C	Irinotecan	Busulfan	Methotrexate	VP-16
1/10	0.4 µM	0.05 mM	0.04 mM	1.64 mM	0.0497 mM	0.12 mM	2.2 µM	0.0339 mM
1/100	0.04 µM	0.005 mM	0.004 mM	0.164 mM	0.0049 mM	0.012 mM	0.22 µM	0.0033 mM
1/1000	0.004 µM	0.0005 mM	0.0004 mM	0.0164 mM	0.00049 mM	0.0012 mM	0.0022 µM	0.00033 mM
**Clinical dose**	10 mg/Kg	75 mg/m∧2	80 mg/m∧2	2 g/m∧2	125 mg/m∧2	150 mg/m∧2	5 mg/m∧2	100 mg/m∧2
**Calculated in vitro dose equivalent to clinical dose**	**4 µM**	**0.5 mM**	**0.4 mM**	**16.44 mM**	**0.497 mM**	**1.22 mM**	**22 µM**	**0.3397 mM**

Calculated in vitro dose  =  [(clinical dose in mg/m∧2×2 m∧2)/1000]/MW of drug.

Sensitivity to chemotherapy was assessed at 48-hours by WST8 viability assay. It was categorized as follows based on the percentage of non-viable cells: responsive (0–40% cell survival), intermediate (40–70% cell survival), and non-responsive (70–100% cell survival). The WST8 assay was conducted three separate times with n-5 well replicas/drug/dose each time.

**Figure 4:**
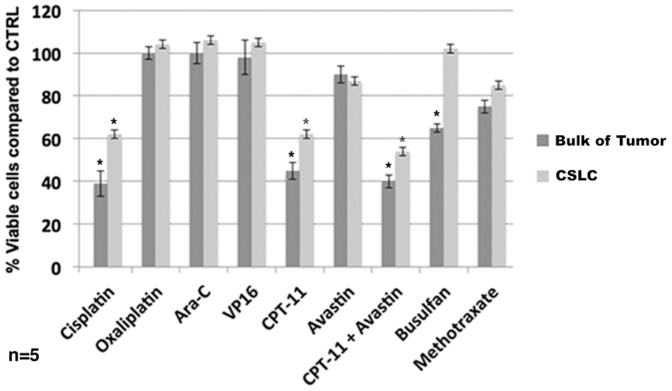
Diagram of ChemoID Assay to Assess the Sensitivity to Chemotherapy of Cancer Cells or CSLCs Using a WST-8 Assay on patient 1. 1×10∧3 bulk of tumor cells or CSLCs plated in 5 replicas into 96-well plates were challenged for a 1-hour pulse with a panel of anticancer drugs indicated by the oncologist. A WST-8 assay was performed 48-hours following chemotherapy treatments to assess cell viability. Data is plotted in bar graph as responsive (0–40% cell viability), moderately responsive (40–70% cell viability), and non-responsive (70–100% cell viability). Light grey bar represent sensitivity of CSLCs to chemotherapy with respect to negative untreated control cells. Dark grey bar represent sensitivity of bulk of tumor cells to chemotherapy with respect to negative untreated control cells. Anticancer drugs tested indicated at the bottom of the diagram. Statistical analysis of the significance of the results was performed with a 1-way ANOVA. Asterisks indicate *p* values of less than 0.05.

ChemoID assay showed that the ependymoma cells grown in monolayer and representing the bulk of tumor cells were sensitive to clinically relevant doses of cisplatin, irinotecan, busulfan, and a combination of irinotecan and bevacizumab in a statistically significant manner (p<0.05). Interestingly, the CSLCs were sensitive to a combination of irinotecan and bevacizumab (p<0.05), intermediately sensitive to cisplatin, and irinotecan, but not sensitive to busulfan. On the other hand, both the CSLCs and the bulk of tumor cells were not responsive to methotrexate, oxaliplatin, arabinoside-C, and VP-16 (**Figure 4**).

Because of the lack of response to an oxaliplatin and etoposide management given in August 2009 (**Figure 2B**) (which was started prior to receiving the results from the ChemoID assay), the patient underwent in October 2009 a treatment with bevacizumab and irinotecan, which was administered every two weeks for 6 months. In a follow-up MRI scan in May 2010 the patient showed initial disease regression remaining free from disease progression for 18 months (**Figure 2 C and D**). This corresponded to the longest disease progression free period observed in this patient without major de-bulking surgery.

Recurrence of tumor growth after 18 months of disease free progression led us to explore novel therapeutic approaches for the treatment of this patient's cancer. In this regard, combination chemotherapy was investigated in order to identify natural compounds that may increase the clinical efficacy of anticancer drugs.

**Figure 5:**
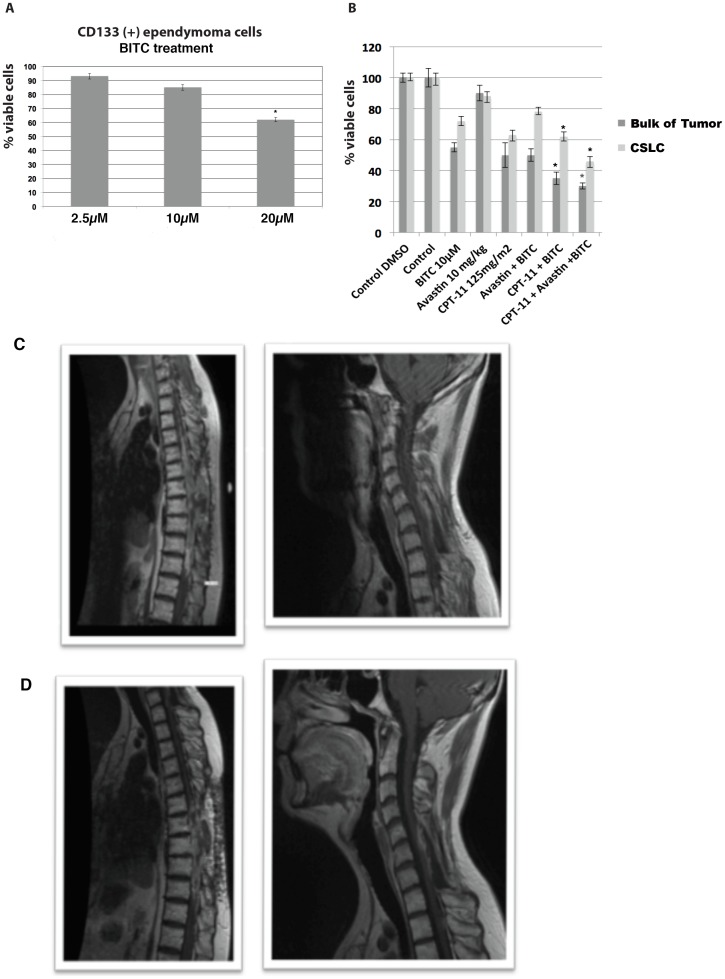
Diagram of ChemoID Assay and MRI Images of Cervical and Thoracic Spine following Integrated Therapy. **A**) 1×10∧3 CSLCs plated in 5 replicas into 96-well plates were challenged for a 1-hour pulse with 2.5, 10, and 20 µM BITC. A WST-8 assay was performed 48-hours after treatments to assess cell viability. **B**) 1×10∧3 CSLCs plated in 5 replicas into 96-well plates were challenged for a 1-hour pulse with 10 µM BITC followed by a 1-hour pulse with 0.5 mM CPT-11. A WST-8 assay was performed 48-hours following chemotherapy treatment to assess cell viability. Data is plotted in bar graph as responsive (0–40% cell viability), moderately responsive (40–70% cell viability), and non-responsive (70–100% cell viability). Light grey bar represent sensitivity of CSLCs to chemotherapy with respect to negative untreated control cells. Dark grey bar represent sensitivity of bulk of tumor cells to chemotherapy with respect to negative untreated control cells. Statistical analysis of the significance of the results was performed with a 1-way ANOVA. Asterisks indicate *p* values of less than 0.05. **C**) 2012 MRI of the cervical and thoracic spine showing recurrence after an 18 months progression free period. **D**) 2012 MRI of the cervical spine showing marked tumor regression of the thoracic spine lesion following combined treatment with irinotecan (CPT11), bevacizumab (Avastin), and BITC supplementation.

BITC has been shown in other laboratories [14], [15] to increase the chemosensitivity of cancer cells. We have recently observed in our laboratory (data not shown) that benzyl isothiocyanate (BITC) increases specifically the chemosensitivity of CD133 positive cancer cells. Because the primary ependymoma cells of our patient displayed a high percentage of cells positive to CD133, we wanted to test the hypothesis that BITC could increase their chemosensitivity to irinotecan and bevacizumab. We found with the ChemoID assay that increasing concentrations of BITC ranging from 2.5 µM to 20 µM decreased the viability of CD133(+) ependymoma cells of Patient 1 from 90% to 62% in a statistically significant manner (**Figure 5A**). ChemoID assay also determined that the combination of irinotecan and a non-toxic concentration of 10 µM BITC reduced the viability of the ependymoma cells from 60% to 40% (over 40% more chemosensitive compared to non BITC treated cells) (**Figure 5 B**). Additionally, the combination of irinotecan and bevacizumab with BITC reduced even further the viability of the ependymoma cells to 30% (**Figure 5 **B). The patient was treated with irinotecan and bevacizumab, but this time with the combination of 2 capsules/day of a Triple Action Cruciferous Vegetable Extract containing high concentration of BITC (LifeExtension, http://www.lef.org), for two months. Following the combination therapy of irinotecan, bevacizumab and the supplement of cruciferous vegetables, we have observed a 4 cm regression (which corresponds to a 50% regression) of the lesions in the thoracic and the cervical area [compare **Figure 5C** (at recurrence) to **Figure 5D** (following therapy)]. Additionally, we report that the patient was able to tolerate the entire course of irinotecan and bevacizumab chemotherapy regimen with less fatigue and tolerance to cold.

**Figure 6:**
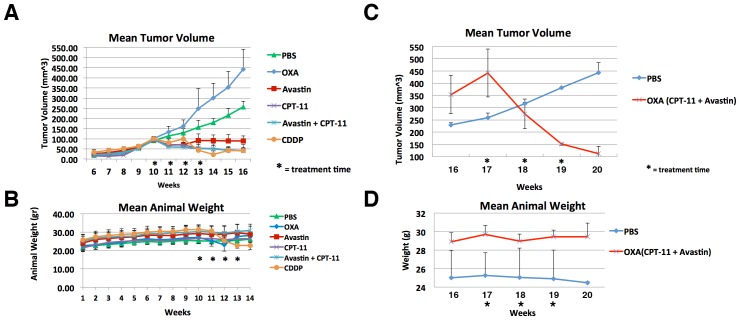
Mean Tumor Volume and Mean Tumor Weight of Patient Derived Xenografts Treated with i.p. Injection of Anticancer Drugs. A) Line diagram of the mean volumes in mm∧3 (±SD) from week 6–16 of 10 patient derived xenografted tumors in NOD-Scid mice following 4 weeks of treatment with various anticancer drugs. The mean tumor volumes are indicated on the ordinate. Asterisks indicate weeks in which treatment was performed. On the right are indicated the different treatment arms. PBS: saline solution, negative control. OXA (oxaliplatin); Avastin (bevacizumab); CPT-11 (irinotecan); CDDP (cisplatin). B) Line diagram of the mean weight in grams (±SD) of 10 NOD-Scid mice-bearing patient derived xenografted tumors following 4 weeks of treatment with various anticancer drugs. The mean tumor weights are indicated on the ordinate. Asterisks indicate weeks in which treatment was performed. On the right are indicated the different treatment arms. PBS: saline solution is negative control. OXA (oxaliplatin); Avastin (bevacizumab); CPT-11 (irinotecan); CDDP (cisplatin). C) Line diagram of the mean volumes in mm∧3 (±SD) from week 16 to 20 of the 10 patient derived xenografted tumors in NOD-Scid mice that failed oxaliplatin therapy (weeks 6–16 in panel A), following 3 weeks of treatment with irinotecan and bevacizumab. The mean tumor volumes are indicated on the ordinate. Asterisks indicate weeks in which treatment was performed. On the right are indicated the different treatment arms. PBS: saline solution, negative control. OXA (CPT11+Avastin): mice that failed oxaliplatin and were then treated with irinotecan and bevacizumab. D) Line diagram of the mean weight in grams (±SD) of the 10 NOD-Scid mice-bearing patient derived xenografted tumors following 3 weeks of treatment with irinotecan and bevacizumab. The mean tumor weights are indicated on the ordinate. Asterisks indicate weeks in which treatment was performed. On the right are indicated the different treatment arms. PBS: saline solution, negative control. OXA (CPT11+Avastin): mice that failed oxaliplatin and were then treated with irinotecan and bevacizumab.

The efficacy of chemotherapies screened in vitro by the ChemoID assay were tested on the ependymoma cells of Patient 1 that were xenografted in a NOD-*Scid* mouse model (**Figure 6** **A and B**). Ten athymic NOD-Scid mice were injected in the flank with 1×10∧6 ependymoma cells mixed to 100 µL of matrigel (BD Biosciences, San Jose, CA) and tumors were grown for 10 weeks or until 100 mm∧3. Randomized mice were treated by weekly intraperitoneal (i.p.) injections of the different treatment arms for 4 weeks and were observed for 4 more weeks. Group #1 serving as a control received i.p. sterile saline injections. Groups #2–5 were the experimental groups, which received i.p. injections of the least effective chemotherapy, or the most effective, the second most effective, and the most effective combinatorial chemotherapy, as determined by the *in vitro* ChemoID assay.

Interestingly, the tumor xenografts in the Scid mice injected with the least effective chemotherapy as determined by the *in vitro* ChemoID assay grew faster than saline control injected mice (**Figure 6A**). As expected, we observed tumor regression in Scid mice treated with the most effective, the second most effective, and the most effective combinatorial chemotherapy as determined by the *in vitro* ChemoID assay, confirming the clinical observation that irinotecan and bevacizumab are more effective anticancer drugs in this individual patient. Mice weight was measured weekly (**Figure 6B**)

We further tested the hypothesis that mice that were failing a chemoresistant treatment could be rescued by switching them to a more sensitive treatment as determined by the *in vitro* ChemoID assay. Mice that were failing an oxaliplatin therapy regimen were taken off oxaliplatin at week 16 and were treated for 4 weeks with a combination of irinotecan and bevacizumab. As expected, mice treated with irinotecan and bevacizumab showed a regression of the xenografted tumor compared to the control mice injected with saline solution (**Figure 6C**) confirming once again the previously observed clinical data.

### Patient 2 History and ChemoID Results

Patient 2 is a 5-month-old female with an aggressive brain tumor that was surgically removed in April 2012. The tumor was diagnosed as an anaplastic ependymoma, WHO grade III with low-grade mitosis-poor areas and high cellular tissue with mitosis and high MIB-1 rate.

A biopsy from the surgically removed tumor was placed in RPMI-1640 sterile media and the tissue was dissociated in our laboratory into a single-cell suspension with the use of a GentleMACS tissue dissociator (Miltenyi, Aubourn, CA) as previously. The single-cell ependymoma suspension was plated in RPMI-1640 in the presence of 5% irradiated, heat inactivated, defined fetal bovine serum, streptomycin and penicillin and cells were cultured as a monolayer for 15 days. Cells were immunophenotyped by flow cytometer using antibodies against CD34, CD38, CD44, CD133, Nanog, and CXCR4. The ependymoma cells were found positive to Nanog (13%), CD133 (47.5%), CD44 (65.5%), and CXCR4 (89.7%) when compared to an isotype control antibody. A double staining of CD34 and CD38 showed the presence of 4.6% of the cells CD34+/CD38+, and 47.3% CD34+/CD38- cells (data not shown).

**Figure 7:**
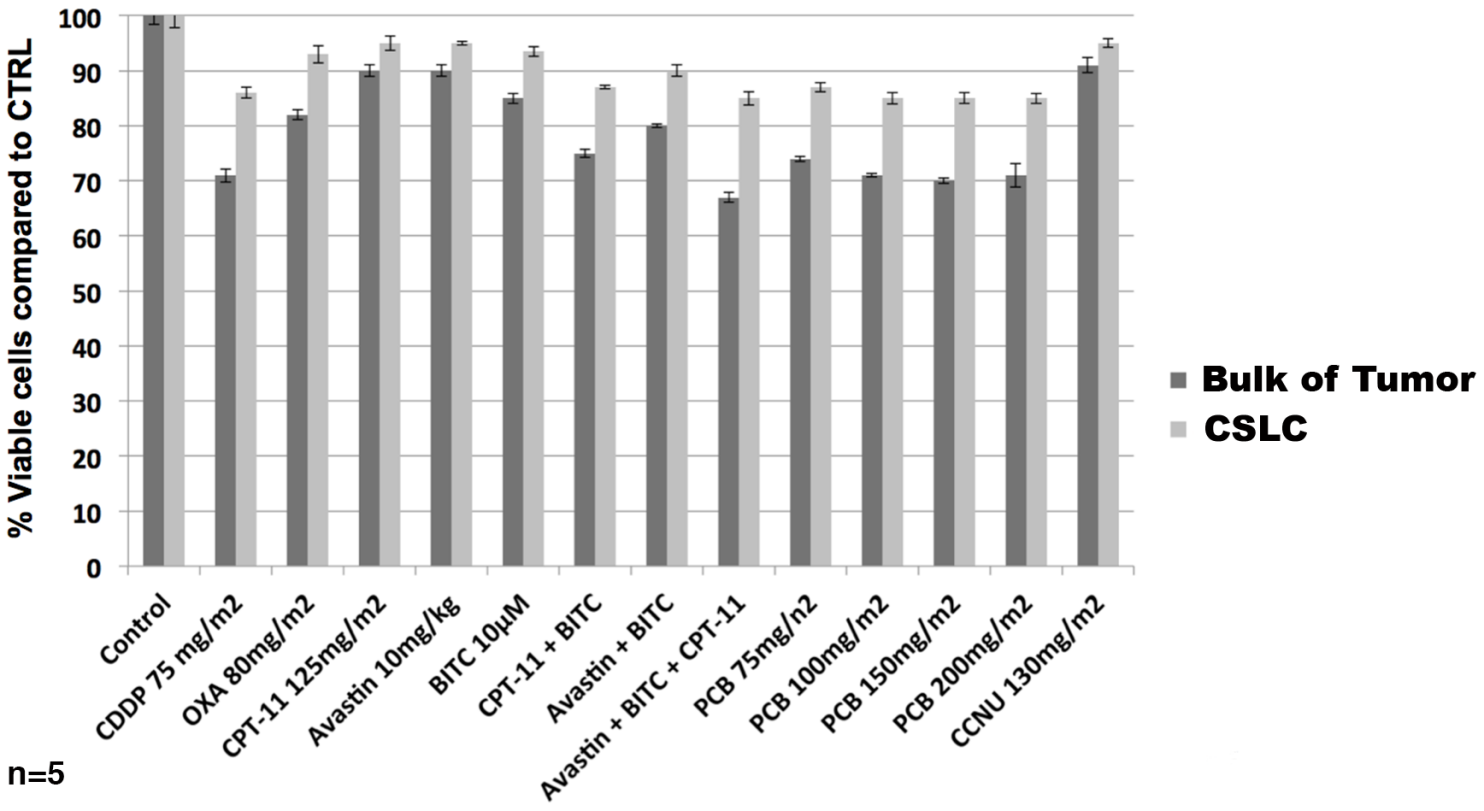
Diagram of ChemoID Assay to Assess the Sensitivity to Chemotherapy of Cancer Cells or CSLCs Using a WST-8 Assay on patient 2. 1×10∧3 bulk of tumor cells or CSLCs plated in 5 replicas into 96-well plates were challenged for a 1-hour pulse with a panel of anticancer drugs indicated by the oncologist. A WST-8 assay was performed 48-hours following chemotherapy treatments to assess cell viability. Data is plotted in bar graph as responsive (0–40% cell viability), moderately responsive (40–70% cell viability), and non-responsive (70–100% cell viability). Light grey bar represent sensitivity of CSLCs to chemotherapy with respect to negative untreated control cells. Dark grey bar represent sensitivity of bulk of tumor cells to chemotherapy with respect to negative untreated control cells. Anticancer drugs tested indicated at the bottom of the diagram. Statistical analysis of the significance of the results was performed with a 1-way ANOVA. Asterisks indicate *p* values of less than 0.05.

The ChemoID assay performed on the bulk of the ependymoma cells and on the CSLCs showed resistance to all of the tested chemotherapy drugs (**Figure 7**). Patient 2 received complex chemotherapy with 6 cycles of vincristine, carboplatin, cyclophosphamide, etoposide, and cisplatin in various combinations, however the tumor rapidly progressed and proton beam therapy was recommended. Because of the lack of tumor response to the various anticancer drugs and radiation therapy, the patient expired after 9 months.

## Discussion

Treatment for ependymoma is often a combinatorial approach that includes surgery, radiation therapy, and chemotherapy. Although chemotherapy has been used extensively in the treatment management of ependymomas, this therapeutic modality is often reserved for patients with residual tumor after surgery and for children younger than 3 years of age in an attempt to delay radiation therapy. Recently, the role of chemotherapy in the treatment of ependymoma has diminished because (1) chemotherapy fails to delay radiation therapy for a meaningful period of time; (2) tumors that progress during chemotherapy do not respond as well to subsequent irradiation; and (3) the combination of chemotherapy and irradiation does not improve overall survival [16].

It is not entirely clear why there is not an improved survival with chemotherapy [5], therefore investigation and development of novel strategies and integrated therapies are required to find more effective treatments for this type of tumor.

One of our patients was diagnosed with recurring undifferentiated intradural-extramedullary spinal ependymoma, WHO grade III, with a distinctive sensitivity to chemotherapy who has been followed up for 5 years following ChemoID. The second patient was also diagnosed with recurring ependymoma, WHO III but was found not sensitive to any of the chemotherapies tested and rapidly progressed.

Resistance to chemotherapy severely compromises its effectiveness. The development of resistance is a major problem for patients, researchers, and clinicians who rely on conventional cytotoxic agents for the treatment of cancer.

Despite the fact that several treatments for ependymoma are currently available, this remains a poorly treated disease [17]–[21]. Surgery plus postoperative radiotherapy represents the standard treatment for patients with grade III (anaplastic) ependymomas [21], [22]. Additionally, surgery has been demonstrated to be associated with significant improvements in overall survival time for patients with all stages of ependymal tumors [23]–[27]. However, a total resection is not always achieved. Overall prognosis is improved when the entire tumor can be removed and there are no other neural axis metastasis [28]. Therefore, in cases in which the ependymoma is multifocal, metastatic, incompletely resected, or particularly aggressive; it is imperative to find the most effective alternative treatment to surgery available.

Administration of ineffective anticancer therapy is associated with unnecessary toxicity and development of resistant clones. Each time patients are treated, they have a chance of relapse and their cancer may become more resistant to therapy. Presently used anticancer drugs have a high rate of failure and cell culture chemotherapy testing is being used to identify which drugs are more likely to be effective against a particular tumor type. Measuring the response of the tumor cells to drug exposure is valuable in any situation in which there is a choice between two or more treatments. This includes virtually all situations in cancer chemotherapy, whether the goal is cure or palliation. This kind of testing can assist in individualizing cancer therapy by providing information about the likely response of an individual patient's tumor to proposed therapy. Many attempts have been made over the years to develop an *ex-vivo* anti-cancer test that can provide clinically relevant treatment information, but all the efforts have been directed toward the bulk of tumor cells [29]–[35].

In the recent past, chemotherapy testing has been performed on cancer cells from patients without prior separation and enrichment of the CSLCs from the bulk of tumor cells [30], [36]–[45].

Knowing which chemotherapy agents the patient's bulk of tumor cells as well as the CSLCs are resistant to is very important. Then, these options can be eliminated, thereby avoiding the toxicity of ineffective agents. Choosing the most effective agent can help patients to avoid the physical, emotional, and financial costs of failed therapy and experience an increased quality of life.

ChemoID chemotherapy sensitivity assay used in this study, measures for the first time the survival of CSLCs and bulk of tumor cells cultured from human cancer biopsies following chemotherapy. The advantage of the ChemoID assay is to aid the oncologists in selecting the most appropriate chemotherapy regimen on an individual basis especially when a number of equivalent options are available. The ChemoID assay allows various available chemotherapy drugs, which are part of standard of care to be tested, for efficacy against the cancer stem cells as well as the bulk of tumors.

For patient 1 affected by a recurring anaplastic ependymoma, the ChemoID assay determined on both bulk of tumor cells and CSLCs, that the most effective treatments were either irinotecan and bevacizumab or cisplatin. Interestingly, although the entire regimen containing irinotecan and bevacizumab could not be completed, the patient showed an initial regression of the disease and remained free from disease progression for 18 months, which corresponded to the longest disease progression free period in this patient.

Following up on the recurrence after the 18 month of progression free interval observed, repeated testing was performed using the ChemoID assay on the combination of several drugs and nutritional supplements among which benzylisothiocyanate (BITC). Numerous studies have indicated that isothiocyanates (ITCs) induce robust anti-cancer effects [15], [46], [47]. ITCs are derived naturally from glucosinolates, which are found at high concentrations in vegetables from the Cruciferae family [14], [15]. Cruciferous vegetables, which produce ITCs, include broccoli, Indian cress, cabbage, Brussel sprouts, and watercress [48]. ITCs are of interest as anticancer molecules because of their ability to target many of the aberrant pathways associated with cancer development. However, among the numerous ITCs identified, only a few of them appear to elicit anti-carcinogenic properties [49].

Interestingly, BITC has been previously shown to increase the chemosensitivity of bulk of tumor cells [14], [15], but not of CSLCs. In our laboratory we have observed that BITC can increase specifically the chemosensitivity of cells that are highly positive for CD133 (data not shown), a marker used to identify CSLCs in tumors of the nervous system. Since the primary ependymoma cells of our patient displayed a high percentage of cells positive to CD133, we tested the hypothesis that BITC could increase their chemosensitivity.

Interestingly, we demonstrated here, for the first time, that the combination of irinotecan and BITC increased the chemosensitivity of the bulk of tumor cells and of the CSLCs cultured from the ependymoma of patient 1and have observed a clinically significant regression of the lesion in the cervical area as well as regression of other lesions at the thoracic level following a combined treatment with irinotecan, bevacizumab, and BITC.

Noteworthy and as expected, we observed regression of the NOD-Scid mice xenografts treated with the most effective, the second most effective, and the most effective combinatorial chemotherapy as determined by the *in vitro* ChemoID assay. In a model of patient derived xenografts this confirms the clinical observation that irinotecan and bevacizumab are more effective anticancer drugs for this individual patient. Interestingly, the tumor xenografts in the Scid mice injected with the least effective chemotherapy as determined by the *in vitro* ChemoID assay grew faster than saline control injected mice. We do not know why the tumor xenografts in mice injected with oxaliplatin grew faster than saline control injected mice, but we speculate that because the patient was treated with oxaliplatin prior to the ChemoID assay biopsy, it had selected cellular clones that are resistant to it and that manifest a growth advantage in its presence.

Furthermore, mice that failed to oxaliplatin treatment, which mimics the clinical scenario of this particular patient, were rescued by switching them to a more sensitive treatment (irinotecan and bevacizumab) as determined by the *in vitro* ChemoID assay. As expected, in this rescue animal model the mice treated with a combination of irinotecan and bevacizumab showed a regression of the patient derived xenografted tumors compared to control mice injected with saline solution confirming once again the previously observed clinical data.

Unfortunately, the second case of ependymoma we present could not benefit from any combined therapy that was proposed indicating that although affected by the same type of tumor response to chemotherapy can be different.

This is the first report on the clinical relevance of this novel chemosensitivity assay that measures the sensitivity of bulk of tumor cells and CSLCs to chemotherapy, which has the objective to decrease unnecessary toxicity while increasing the benefit of cytotoxic therapy for patients affected by malignant tumors.

Although the ChemoID results on these two cases of ependymoma showed clinical relevance, a larger study with different histological tumor types is needed to determine the prognostic accuracy of this assay. We are currently conducting a brain and spine malignant tumor phase-I clinical trial in which we have accrued 33 patients in the past three years to study the feasibility of this new assay in predicting the most effective chemotherapy regimen to improve patients' outcomes by assessing the vulnerability to chemotherapy of the CSLCs.

## Disclosures

All research involving human participants has been approved by the authors' institutional review board, protocol #326290. Informed consent was obtained and all clinical investigation was conducted according to the principles expressed in the Declaration of Helsinki.

All animal work was conducted according to relevant national and international guidelines. All animal studies have been conducted following approval from the Marshall University IACUC, protocol #373017.

## Supporting Information

Figure S1 **Characterization of the Primary Ependymoma Cell Culture and of the Enriched CSLCs.** ***A-F)*** *Immunophenotype conducted using*: **A)** OCT3/4 antibody; Left panel: isotype antibody (bulk of tumor cells); Center panel: specific antibody (bulk of tumor cells); Right panel: specific antibody (enriched CSLCs). **B)** Nanog antibody; Left panel: isotype antibody (bulk of tumor cells); Center panel: specific antibody (bulk of tumor cells); Right panel: specific antibody (enriched CSLCs). **C)** CD133 antibody; Left panel: isotype antibody (bulk of tumor cells); Center panel: specific antibody (bulk of tumor cells); Right panel: specific antibody (enriched CSLCs). **D)** CD117 antibody; Left panel: isotype antibody (bulk of tumor cells); Center panel: specific antibody (bulk of tumor cells); Right panel: specific antibody (enriched CSLCs). **E)** CD44 antibody; Left panel: isotype antibody (bulk of tumor cells); Center panel: specific antibody (bulk of tumor cells); Right panel: specific antibody (enriched CSLCs). **F)** Double labeling with CD34 and CD38 antibodies; Panel on left: isotype antibody (bulk of tumor cells); Center panel: specific antibody (bulk of tumor cells); Panel on right: specific antibody (enriched CSLCs).
(TIF)
