# Supplementary figures and images for: Chemo-Predictive Assay for Targeting Cancer Stem-Like Cells in Patients Affected by Brain Tumors
Source: PLoS One. 2014 Aug 21;9(8):e105710. doi: 10.1371/journal.pone.0105710 (PMC4140819; doi:10.1371/journal.pone.0105710)

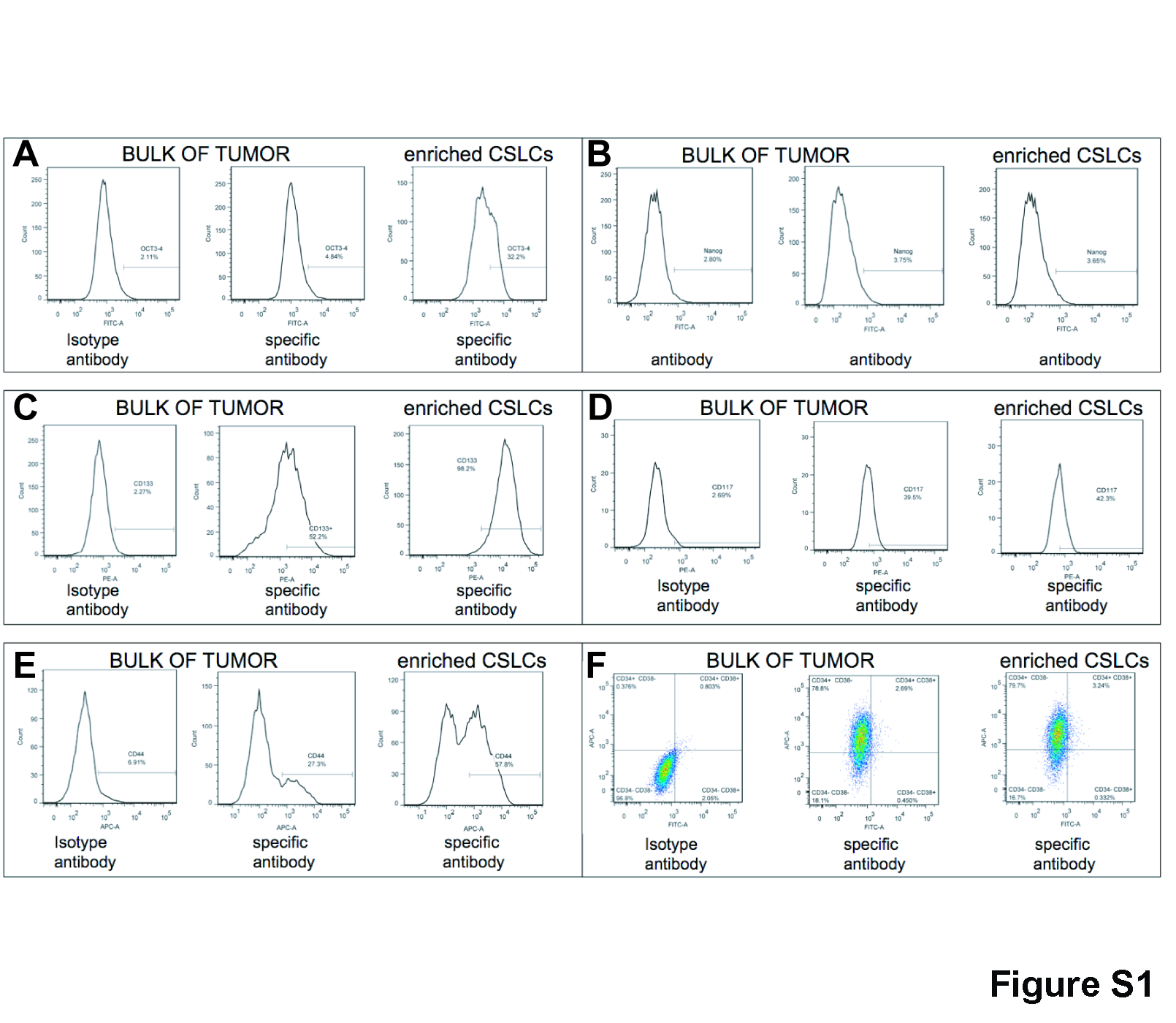

Supplement: Figure S1 — Characterization of the Primary Ependymoma Cell Culture and of the Enriched CSLCs. A-F) Immunophenotype conducted using: A) OCT3/4 antibody; Left panel: isotype antibody (bulk of tumor cells); Center panel: specific antibody (bulk of tumor cells); Right panel: specific antibody (enriched CSLCs). B) Nanog antibody; Left panel: isotype antibody (bulk of tumor cells); Center panel: specific antibody (bulk of tumor cells); Right panel: specific antibody (enriched CSLCs). C) CD133 antibody; Left panel: isotype antibody (bulk of tumor cells); Center panel: specific antibody (bulk of tumor cells); Right panel: specific antibody (enriched CSLCs). D) CD117 antibody; Left panel: isotype antibody (bulk of tumor cells); Center panel: specific antibody (bulk of tumor cells); Right panel: specific antibody (enriched CSLCs). E) CD44 antibody; Left panel: isotype antibody (bulk of tumor cells); Center panel: specific antibody (bulk of tumor cells); Right panel: specific antibody (enriched CSLCs). F) Double labeling with CD34 and CD38 antibodies; Panel on left: isotype antibody (bulk of tumor cells); Center panel: specific antibody (bulk of tumor cells); Panel on right: specific antibody (enriched CSLCs). (TIF) [file pone.0105710.s001.tif]
